# On the Medicinal Effects of the Salt of Tartar, in Puerperal Fevers, &c.

**Published:** 1800-03

**Authors:** 


### *On the Medicinal Effects of the Salt of Tartar, in Puerperal Fevers, &c.*

[ Continued from our last Number, pp. 165—167. ]

### Observation I.

The female Cit. Letourneur, aged 24, having been delivered five days, and made unsuccessful attempts to suckle her child, was so much afflicted that she became extremely indisposed. Cit. Guinot found her pulse contracted, hard, and very frequent. Her breasts were flaccid; the hypogastric region was inflated, and the whole abdomen painful. The lochia ceased; the patient complained of a temporary blindness, was quite deaf, and became subject to violent and continual delirium. Cit. Guinot applied leeches to the vagina, and prescribed fifteen grains of the carbonat of potass, in a potion of four ounces of lilly flower water, with an ounce of syrup of orange-flowers, a spoonful to be taken every hour; during a temporary remission, he applied blisters to her legs. The lochia again appeared; the symptoms diminished by degrees; the patient continued the carbonat of potass for three weeks; she was then purged, and the cure was completed.

### Observation II.

On the 9th Ventose, sixth year, the female Citizen Rebours, thirty years of age, of a bilious, vigorous, and irritable temperament, had a very difficult parturition, which, after two days painful labour, terminated by the aid of the forceps. Shortly after, she experienced very acute pains in the abdomen, accompanied by a severe, dry, and frequent cough. At length, a fever intervened, the pulse became hard and bound, and the lochia almost entirely ceased. Diluents and composing remedies were employed without success. About the fourth day, a slight swelling of the breast afforded some hopes of relief, but the milk soon disappeared; the breasts were flaccid, the lochia suppressed, the abdomen, and particularly the hypogastric region, considerably tumefied; her sufferings became more distressing, her cough more obstinate; and the patient was attacked with delirium. On the sixth day, a miliary eruption appeared with spots of a red-violet colour. At this period, Cit. Guinot had recourse to anti-lacteal remedies. He prescribed fomentations, composed of a solution of soap in a decoction of poppy heads, to be applied to the abdomen; a potion of the carbonat of pot-ass, at the rate of twelve grains to a dose, to be taken by a ſpoonful at a time; and an emollient clyſter to be adminiſtered morning and evening, to which was added a drachm of the carbonat of ſoda. He alſo ordered a dozen leeches to be applied to the vagina; the bleeding was abundant, and apparently relieved the patient. The fever, however, increaſed, while the miliary eruption and ſpots diſappeared; they were ſucceeded by tranſparent veſicles of the ſize of a pea, ſimilar to thoſe which riſe in *pemphigus.* Theſe ſymptoms announced a great tendency to putrefaction. The alcaline treatment was conſequently ſuſpended, and the Virginian ſnake root, a potion made of bark, a light diaphoretic drink, with compoſing clyſters, and antifebrile remedies, were preſcribed. On the twelfth day, the eruption diſappeared, leaving on the ſkin a furfuraceous duſt. The fever, delirium, and cough, as well as all the other ſymptoms, ſtill remained; but the lochia had entirely diſappeared. The former treatment was again adopted, by increaſing the quantity of the carbonat of potaſs in the draughts, and the carbonat of ſoda in the clyſters and fomentations, while the patient's diet was low. To prevent the conſequences of the ceſſation of the miliary and cryſtalline eruptions, he took the earlieſt opportunity of applying two ſmall bliſters to the legs. From the time the former treatment was adopted, the patient continued to recover; the tenſion of the abdomen, and the other ſymptoms, gradually diminiſhed; the lochia again appeared, and continued to flow; the urine and ſtools, which had hitherto been very ſparing, became more abundant, and had a proper appearance. On the twenty-ſecond day, the patient was able to take a purgative, which operated three or four times. The alcaline treatment was continued till the 20th Germinal, at which time the patient was perfectly cured.

### Observation III.

The female Citizen Lepitre, about thirty years of age, tall, and of a robuſt conſtitution, who had recently been delivered of a dead child, ſent for Cit. Guinot, who found her in the moſt deplorable ſtate. The lochia had ſtopt; the breaſts were flaccid, though tumefied; the belly diſtended and painful; reſpiration very difficult; the voice hoarſe; the ſpeech haſty; the pulſe frequent and hard; the face inflamed, and the eyes ſparkling. From the combination of theſe ſymptoms, he determined upon applying leeches to the vagina; which were attended with a favourable effect. He then preſcribed the carbonat of potaſs in a doſe of from thirty to thirty-ſix grains every day; bliſters were applied to the legs, and ſome purgatives at length terminated the diſeaſe, in the ſpace of a month. Cit. Devilliers aſſiſted Cit. Guinot in the treatment of this diſeaſe.

OBSERVATION IV.

On the night of the 10th of Auguft, 1792, the female Cit. Bourrée, thirty-two years of age, of a ftrong conftitution, was affrighted by the *tocfin* and the *generale*, two or three days previous to her fafe delivery of twins. Her milk difappeared; the abdomen became inflated, and the region of the uterus was extremely painful; the lochia were fuppreffed, an acute fever appeared, which was attended with a flight delirium. Cit. Guinot, who was called in at midnight, immediately prefcribed a potion compofed of four ounces of barley water, half an ounce of orange flower water, half an ounce of fyrup of *karabé*, and eighteen grains of carbonat of potafs; a fpoonful to be taken every half hour. He ordered flannels foaked in hot foap-water, to be applied to the hypogaftric region, and prefcribed clyfters of a fimilar folution. About twelve o'clock on the fame day, the patient felt relieved; but was in great anxiety about her hufband, who was then in the action. She was delirious, and the fever ftill remained, though the lochia in a flight degree returned. Leeches were now applied, and attended with confiderable effect. Her hufband returned; the alcaline treatment was continued; her ftate of health improved; the lochia flowed abundantly; and the patient was cured in the fpace of a fortnight.

OBSERVATION V.

In the month of Floreal, 5th year, the female Cit. Lamarre, twenty-fix years of age, of a middle ftature, but very robuft, having loft the child fhe fuckled, fuddenly experienced a violent attack. Her neck, head, and face, became fwollen and painful; the glands of the neck and the furrounding cellular texture were bloated, and the patient difcharged a milky fluid from the nofe. Thefe fymptoms were attended with a continued and high fever. The metaftafis of the milk could not be prevented, and this cafe excited the attention of Cit. Guinot. The remedies he prefcribed, were clyfters rendered active by the carbonat of foda, in dofes of one drachm each; baths for the feet, and a draught acidulated by the addition of carbonat of potafs. He foon had reafon to be fatisfied with this treatment: The milky difcharge from the nofe ceafed, and was fucceeded by an abundant flow of the fame nature from the vagina. All the fymptoms now abated, and the patient, from difguft, left off her alcaline drink for feveral days, in confequence of which the fymptoms again returned. It was now neceffary to repeat the carbonat of potafs in ftronger dofes; to apply leeches, and direct the ufe of purgatives. After this treatment had been purfued for a month, the cure was accomplifhed.

This

This woman was delivered the following year at St. Germain, and returned to Paris afflicted with fever and inexpressible pains over the whole extent of the pelvis. After having in vain attempted all other means of relief, Cit. Guinot prescribed alkaline fomentations to be applied to the abdomen; clysters and injections of a similar nature into the uterus, and drinks acidulated with eighteen grains of carbonat of potass to each pint. After having pursued this treatment for one month, the patient was perfectly restored.

Cit. Marchais was consulted in this case.

[ To be concluded in our next Number. ]

