# Errata

**Published:** 1800-04

**Authors:** 


P. 266, l. 11, from the bottom; and p. 267, l. 7, from the top, for *acidulated* read *fharpened*.—P. 267, l. 1, of the Note, dele the comma after "ejufdemque."

